# Finding low CHA2DS2-VASc scores unreliable? Why not give morphological and hemodynamic methods a try?

**DOI:** 10.3389/fcvm.2022.1032736

**Published:** 2023-01-04

**Authors:** YiRen Sun, Yunfei Ling, Zijia Chen, Zhengjie Wang, Tao Li, Qi Tong, Yongjun Qian

**Affiliations:** ^1^Department of Cardiovascular Surgery, National Clinical Research Center for Geriatrics, West China Hospital, Sichuan University, Chengdu, Sichuan, China; ^2^West China Medical School/West China Hospital, Sichuan University, Chengdu, Sichuan, China

**Keywords:** atrial fibrillation, left atrial appendage, hemodynamics, morphology, thrombosis, CHA2DS2-VASc score

## Abstract

Patients with atrial fibrillation (AF) suffer from a high risk of thrombosis. Currently, the CHA2DS2-VASc score is the most widely used tool for risk stratification in patients with AF, but it has disappointing accuracy and limited predictive value, especially in those with low scores. Thrombi in patients with AF mostly grow in their left atrial appendages (LAA), which is directly related to the abnormal morphology of the LAA or the left atrium and the unusual hemodynamic state around LAA, which may sensitively evaluate the risk of thrombosis complications in patients with AF and bring bases to clinical plans of medication and operation. Therefore, we investigated the research progress of hemodynamic and morphological studies about the predictive value of thrombosis risk in patients with AF, intending to discuss the prediction potential of morphological and hemodynamic indexes when compared with the presently used CHA2DS2-VASc system and how to build a more precise thromboembolic event prediction model for patients with AF.

## 1. Introduction

Atrial fibrillation (AF) is one of the most common types of arrhythmias. Patients with AF are threatened by a high incidence of thrombosis and lethal complications such as stroke (1). The CHA2DS2-VASc system is the most widely used model to predict the risk of thromboembolic events in patients with AF, while several studies demonstrated a limited predictive value of these traditional models in risk stratification (2–4). Especially in patients with low CHA2DS2-VASc scores, their thrombus risk has always been underestimated, and additional parameters may be essential for more reliable risk prediction (5, 6). Several studies tried some biochemical parameters, such as homocysteine and mean platelet volume, but invasiveness, prohibitive costs, and low availability limited their practicability (6–9). Over 90% of thrombi in patients with AF are formed in the left atrial appendage (LAA) (10), which means that the LAA and the left atrial thrombus (LAT) need to be the focus of attention. Studies showed that abnormal LAA morphology and the hemodynamic parameters affected by it are closely related to thrombus formation (11–14), which may be adequate for risk prediction.

However, many of the studies about LAA morphology or hemodynamics are retrospective, which is not so convincing for their prospective value, while the prospective studies each uses few indicators (15, 16) and are unable to show comprehensive conclusion about whether the morphological or hemodynamic indicators are sufficient or not, let alone the question about which indicators are better for prediction. To answer these questions, we searched PubMed and CNKI for published studies. Then, we performed a comprehensive analysis of those with clear data carried out mainly in the last 5 years focusing on the relationship between morphological or hemodynamic indexes and thrombosis risk in patients with AF and those discussing their predictive value compared with the commonly used CHA2DS2-VASc score. Some of these studies even tried to build new prediction models involving new parameters. We summarized their contribution to predicting the thrombus risk in patients with AF, attempting to discuss whether they are ideal supplements for patients, especially those with low CHA2DS2-VASc scores, thus allowing a better selection of patients suitable for LAA occlusion, a shorter duration of the oral anticoagulation, and consequently a reduction in hemorrhagic events.

## 2. Risk assessment of thrombosis in patients with AF: *Status quo* and challenges

### 2.1. The need for thrombosis risk assessment and present use of CHA2DS2-VASc score

Many clinical works are calling for an accurate predictor to guide their practice. Although all patients with AF at risk of thrombus may choose LAA occlusion, it is far from any kind of panacea. The left auricle is not only a so-called “fatal appendage,” and it has important physiological functions such as the storage of pulmonary circulation of blood and secretion of natriuretic peptide, which means that it cannot be resected at will. Approximately 23.9% of the patients had heart failure after LAA occlusion, and 93.8% of them had an increase in their mitral E/e′, while the patients without heart failure did not have such an increase, which stresses the importance of correctly selecting patients and potential predictive value of hemodynamic parameters for heart failure after LAA occlusion (17). In addition, anticoagulants also have the risk of lethal bleeding complications that cannot be ignored. Low stroke risk in patients with AF is reported to be over-treated (18). Therefore, we need a precise predictor for thromboembolic risk in patients with AF.

**FIGURE 1:**
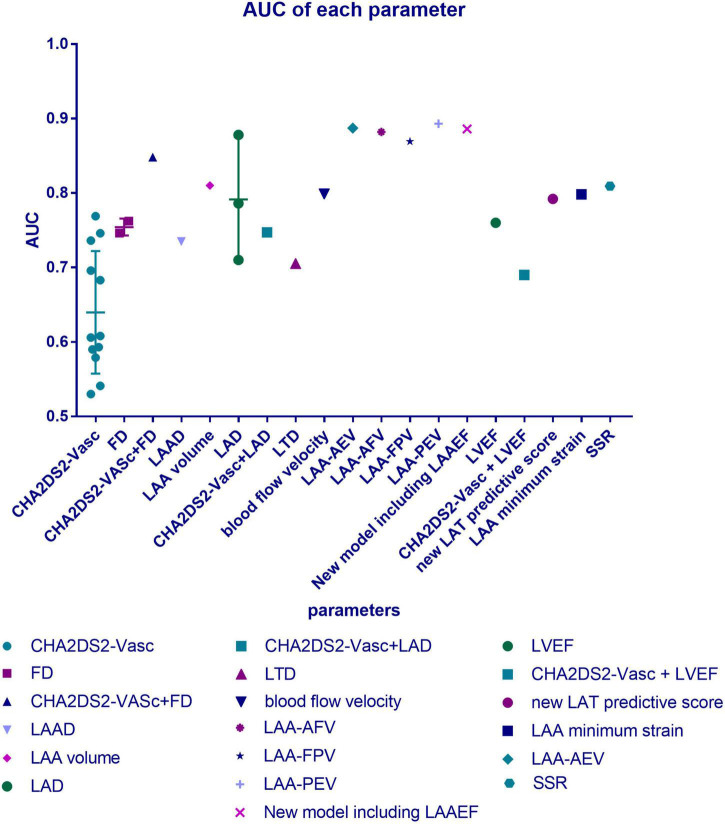
Distribution of area under the ROC curves (AUCs) of the parameters mentioned. Data were collected from numerous studies reporting the parameters’ predictive value for thrombus risk in patients with atrial fibrillation (AF), and this figure illustrates their distribution. The AUC shows the accuracy of a predictive tool. The closer the AUC is to 1.0, the better the predictor is. AUC = 1.0 means that it is a perfect classifier, (0.85, 0.95) shows a very good effect, while (0.7, 0.85) means the effect is proper, and (0.5, 0.7) gets a moderate effect. When it drops to 0.5, it means that the model has no predictive value, and its result is similar to a random guess.

At present, the CHA2DS2-VASc score [previous stroke, 2 points; age: ≥75 years, 2 points; age: 65–74 years, 1 point; congestive heart failure, 1 point; hypertension, 1 point; diabetes mellitus, 1 point; vascular disease, 1 point; sex (female), 1 point] is the most commonly used scoring system for the risk stratification of stroke in patients with AF, and it even exhibits some predictive value in patients with sinus rhythm (19, 20). It has been recommended in the European guidelines as class I for risk stratification in patients with AF. With a CHA2DS2-VASc score of 0 in men or 1 in women, no anticoagulant therapy should be initiated. When the score comes to one in men or two in women, anticoagulation should be considered, weighing bleeding risk against stroke risk. Evidence of a CHA2DS2-VASc score of >2 in men or >3 in women strongly supports the benefit of anticoagulation therapy (10, 21). When used for risk prediction in some cases, the mean CHA2DS2-VASc score was found to be significantly higher in patients with LAT than in patients without LAT, and each additional point comes with a 66% increase in the risk of developing thrombus (22, 23), which means that the CHA2DS2-VASc score is a useful tool for stroke risk prediction in patients with AF. In some studies, this system obtained high areas under the receiver operating characteristic (ROC) curves, higher than 0.7 in a few cases, showing its considerable value in prediction for the entire patients with AF (Figure 1). Moreover, the CHA2DS2-VASc score can be used in many other conditions, for example, assessing the risk of developing prosthetic valve thrombosis in patients with prosthetic valves (24).

However, in clinical practice, it remains controversial whether anticoagulant therapy should be administered in patients with low CHA2DS2-VASc scores (≤1 in men, ≤2 in women) (20), and for patients with hypertrophic cardiomyopathy (HCM) or Friedreich ataxia (25, 26), not excluding many other special conditions, the CHA2DS2-VASc score was found to be useless, and some researchers who noticed these conditions suggested antithrombotic therapy for all of these patients regardless of the CHA2DS2-VASc score. In a retrospective cohort of patients with HCM, 3.6% had a thrombosis event during a 10-year follow-up, while 27.5% of their scores were 0 (27). This will lead to underestimation of low-score patients’ risk and over-treatment of low-risk patients with high scores (18). The risk stratification method used currently is based on demographic variables, while it is not guaranteed to be accurate when applied to individual patients. It assigns points according to existing diseases, (e.g., diabetes and hypertension), age, and gender (16), which are largely retrospective, without taking into account any factors directly related to Virchow’s triad, which may explain why it is recognized that the thrombus risk stratification in patients with AF should not depend solely on factors in the CHA2DS2-VASc score.

### 2.2. The challenges we are facing: The modest prognostic performance of CHA2DS2-VASc

In clinical practice, the usage of the CHA2DS2-VASc score in risk prediction has become unsatisfactory. Many studies found that there is a difference between the score and the patient’s actual thrombosis rate (6, 28). Its prognostic performance of LAT in AF was criticized to be “modest” (20, 29, 30). Particularly in patients with low thrombosis risk, its sensitivity in identifying LAT was judged as “limited” and “unsatisfying” (31, 32). On the one hand, patients with low CHA2DS2-VASc scores have a considerable rate of LAT. The annual stroke rate was reported to be approximately 3% among men whose score was one and among women whose score was two, while the incidence of thrombotic events even reached 11.4% in patients with AF with CHA2DS2-VASc scores of 0 to 1 (33, 34). However, some patients with high scores did not have as high a risk as expected. In one cross-sectional study, among a total of 838 patients, only 132 (15.8%) patients had LAT, while 475 (56.9%) of them got high scores of more than two (29). Some observational cohort studies and multivariate analyses reported that the CHA2DS2-VASc scores were not positively associated with LAT, and there were no significant differences in the CHA2DS2-VASc score between the LAT group and the control group. In addition, sex, hypertension, history of heart failure, history of stroke, and diabetes mellitus were not significant predictors of LAA thrombus (29, 30, 35, 36). In conclusion, in both the high-score group and the low-score group, the result of the CHA2DS2-VASc scale is not so precise, which means that its sensitivity and specificity may not be satisfactory, which illustrates that the moderate area under the ROC curves (AUC) are shown in Table 1. Only a few cases have an AUC of >0.7, which is still lower than other new methods. The C-statistics of it for ischemic stroke only ranged from 0.57 to 0.67 across multiple cohorts (37). In some of these cases, the relatively higher AUC was obtained from a high specificity at the cost of sensitivity, which drops to 10.80% (38).

**TABLE 1:** Data and resources of the parameters mentioned.

Index	Criteria	AUC	Sensitivity	Specificity	OR	Data sources	Outcome indicator/Special factor
CHA2DS2-VASc	2	0.59				(29)	Atrial thrombosis
	2	0.53				(20)	LAA thrombosis
	2	0.606			1.236	(19)	Atrial thrombosis
		0.541				(31)	Atrial thrombosis CHA2DS2-VASc ≤ 1 (female ≤ 2)
		0.736	10.80%	96.40%		(38)	LAA thrombosis
		0.608				(30)	Atrial thrombosis
	2.5	0.746	78.10%	61.20%		(23)	Atrial thrombosis
		0.7689				(32)	LAA thrombosis CHA2DS2-VASc scores = 0 or 1
		0.6958				(32)	LAA thrombosis CHA2DS2-VASc scores = 0 or 1
		0.683				(39)	LAA thrombosis non-vitamin K oral anticoagulants
		0.579				(2)	LAA thrombosis
	At least 3				3.12	(15)	LAA thrombosis treated with OAC
	≥3	0.593	86.50%	32.60%		(36)	Atrial thrombosis
Fractal dimension (FD)		0.7462				(32)	LAA thrombosis
		0.7622				(32)	LAA thrombosis CHA2DS2-VASc scores = 0 or 1
CHA2DS2-VASc + FD		0.8479				(32)	LAA thrombosis CHA2DS2-VASc scores = 0 or 1
LAA orifice area Left atrial appendage depth (LAAD)	>4 cm^2^				10.9 together with LAA flow velocity	(46)	Thromboembolic events CHA2DS2-VASc score of 0 or 1 (except 1 point for female)
	>3.5 cm^2^					(40)	LAA thrombosis
	>23.45 mm	0.735	75.70%	74.90%	4.216	(38)	LAA thrombosis
LAA volume		0.81				(48)	LAA thrombosis
LAA end-systolic volume	>18.45 mL					(47)	LAA thrombosis
LAA end-diastolic volume	>9.49 mL					(47)	LAA thrombosis
Left atrial enlargement (LAE)	Existence				6	(30)	Atrial thrombosis
Left atrial diameter (LAD)	>37.5 mm				2.036	(19)	Atrial thrombosis
	44 mm					(31)	Atrial thrombosis CHA2DS2-VASc ≤ 1 (female ≤ 2)
CHA2DS2-VASc + LAD	43.5 mm	0.71	47.10%	85.80%		(34)	Thromboembolic events CHA2DS2-VASc scores = 0 or 1
	≥44.17 mm	0.786	89.60%	60.90%		(36)	Atrial thrombosis
	>43.5 mm	0.878	84.40%	74.70%		(51)	Atrial thrombosis CHA2DS2-VASc ≤ 1 (female ≤ 2)
	Equal to or above 52 mm				8.54	(15)	LAA thrombosis treated with OAC
		0.747				(31)	Atrial thrombosis CHA2DS2-VASc ≤ 1 (female ≤ 2)
Left atrium top and bottom diameter (LTD)		0.705	75.70%	73.70%		(38)	LAA thrombosis
LAA flow velocity	<40 cm/s				10.9 together with LAA orifice area	(46)	Thromboembolic events CHA2DS2-VASc score of 0 or 1 (except 1 point for female)
Blood flow velocity	42.25 cm/s	0.799	90.60%	70.20%		(23)	Atrial thrombosis
LAA-AEV	<33.0 cm/s	0.887	86.70%	80.00%		(51)	LAA thrombosis
LAA-AFV	<27.5 cm/s	0.882	96.70%	73.30%		(51)	LAA thrombosis
LAA-FPV	<55.4 cm/s	0.869	85.70%	82.80%		(52)	LAA thrombosis
LAA peak emptying velocity		0.893				(48)	LAA thrombosis
LAA-EF	0.2		92.00%	88.00%		(54)	Atrial thrombosis
New model including LAAEF		0.886				(2)	LAA thrombosis
Average residence time	>2 s					(54)	LAA thrombosis
Left ventricle ejection fraction (LVEF) CHA2DS2-VASc with LVEF					0.956	(29)	Atrial thrombosis
	<57%	0.76				(29)	Atrial thrombosis CHA2DS2-VASc = 0 or 1
	≤55%				4.38	(30)	Atrial thrombosis
	Equal to or below 40%				OR 1.55	(15)	LAA thrombosis treated with OAC
		0.69				(29)	Atrial thrombosis
LAT predictive score = 1 (non-paroxysmal AF) + 2 (LVEF% ≤ 55%) + 3 (LAE)		0.792				(30)	Atrial thrombosis
Identification of vortex core	Position of vortex core					(16)	LAA thrombosis
LAA minimum strain		0.798				(48) A	LAA thrombosis
Global 2D-strain	6.00%		66%	67%		(5)	LAA thrombosis
Medial 2D-strain	8.00%		48%	80%		(5)	LAA thrombosis
Apical 2D-strain	6.00%		63%	64%		(5)	LAA thrombosis
Lateral 2D-strain	5.40%		56%	67%		(5)	LAA thrombosis
SSR	below 10 s^–1^					(16)	LAA thrombosis
	<2.80 s^–1^	0.809	75%	82.80%		(52)	LAA thrombosis

The treatment therapies for patients with low-score AF are inconclusive due to the lack of methods to assess their thrombosis risk, which poses a challenge to clinical practice, and for patients with high scores, we still need to refine the stroke risk stratification to avoid over-treatment. Some authors recommend individual assessment for stroke risk in patients with AF to choose the best option customized to their clinical situation (39). To achieve this purpose, we cannot rely only on the present CHA2DS2-VASc scale system, since the rise of LAT in anticoagulated patients with low scores suggested the presence of additional stroke risk factors not included in the CHA2DS2-VASc scale. According to Virchow’s triad, thrombosis is determined by the stasis of blood flow, endothelial injury, and hypercoagulability, which suggests that morphological or hemodynamic parameters may be what we expected. Many morphological or hemodynamic studies based on transesophageal echocardiography (TEE) or computer fluid mechanics showed more sufficient efficacy than the CHA2DS2-VASc score, which can be used to improve the accuracy of the present model. Many parameters, such as strain rate (SSR), blood flow vorticity, and left atrial volume, were found to be independently associated with LAT, providing more accurate predictions, especially for patients with low CHA2DS2-VASc scores (16, 22).

## 3. Summary of commonly used morphological and hemodynamic indexes and their predictive value for thrombosis

### 3.1. Relationship between LAA morphology and thrombosis risk

#### 3.1.1 Qualitative morphological indicators and their predictive value for thrombosis

Many qualitative morphological indicators about LAA’s complexity can contribute to thrombosis, but in our view, there are many limitations to their direct clinical usage in risk assessments. LAA may have changeable multi-lobar morphology, which has been proven to contribute to blood stasis as an independent risk factor for thrombus risk (40). According to its morphological characteristics, LAA was divided into four classical types, namely, chicken wing type, cactus type, windsock type, and cauliflower type (41). LAA morphological classification has different effects on several hemodynamic parameters (42). It does cause differences in thrombus risk, but it is difficult to generalize which type must have the highest risk. At present, in regard to the relationship between LAA morphology and thrombus risk, only one vague conclusion has been relatively widely accepted: “the more complex shape has a higher thrombus risk.” However, the classification criteria, such as the concept of central and accessory lobes, are not always clear and replicable, and they always differ according to observers. Three experts tried to classify the same samples into four classical categories but failed to reach a consensus (40). The subjectivity of the classification criteria, which makes it impossible to reach an objective consensus and the two-dimensional properties of the image on which the classification often depends may explain this divergence (42). Therefore, if we still attempt to use image impression to judge the risk, we can only analyze with a large sample size to establish an atlas that does not depend on the observer (43).

However, if we want to judge LAA’s complexity, fractal geometry theory may be a good helper, which offers a parameter called fractal dimension (FD) that can quantitatively evaluate the complexity and irregularity of an object. The AUC of FD was slightly lower than that of the CHA2DS2-VASc score when testing all of the patients, but for the low-to-moderate risk group, the accuracy of FD was much higher than that of the CHA2DS2-VASc score (0.7622 vs. 0.6958). It is even better that, if we combine FD with CHA2DS2-VASc score to examine the medium or low risk in patients with AF, we will get a high AUC of 0.8479 (32), which saves us from the dilemma of judging thrombosis risk in patients with low-score AF, which means that FD may be a good supplement to the traditional system when we suspect some patients with low scores of certain thrombosis risks, especially when they accept some imaging examinations.

#### 3.1.2 Quantitative morphological indicators and their thrombus-risk predictive value

We found quantitative morphological indicators as much better predictors, although we cannot deny that there is still a long way to go, since many studies are retrospective, without telling us about the accuracy of their indicators. The enlargement of LAA in patients with AF can interfere with the emptying of LAA and finally increase the risk of thrombosis. Therefore, the size of LAA is considered to be a risk factor for thrombosis (44). The opening area and end-diastolic volume of the LAA were found to increase with increasing CHA2DS2-VASc risk score (45). An opening area larger than 3.5 cm^2^ brings higher risk, while the cutoff point was reported to be larger than 4 cm^2^ by another group, and it has an odds ratio (OR) of 10.9 counted together with LAA flow velocity (40, 46). Left atrial appendage depth (LAAD) was found to be related to thrombus risk with an OR of 4.216. Its predictive value is considerable, having an AUC of 0.735, with a sensitivity of 75.7% and a specificity of 74.9%, and the cutoff point was more than 23.45 mm (38). An LAA end-systolic volume and an end-diastolic volume larger than 18.45 and 9.49 mL were believed to be critical values (47). There was a significant increase in thrombus events in patients with LAA volumes larger than 8.6 cm^3^ (45). One study reported an impressive AUC of 0.81 using LAA volume (48), which showed its good value of prediction.

The increase in LAA size is not the only factor leading to the stagnation of flow in LAA. Other quantitative morphological indicators, such as curvature, also play an important role (49). With a long length and large curvature, the LAA will have a high risk of thrombus even if it is defined as a simple shape. However, there is also an LAA with a large curvature that is compensated by a limited length, thus showing a low risk of thrombus (50). Therefore, for so many quantitative and qualitative morphological indicators, we should assess them comprehensively.

To evaluate the risk of LAT, the left atrium (LA) cannot be ignored. Studies on LA are far more sufficient. Left atrial enlargement (LAE) was also found to be related to the formation of thrombi (30). Left atrial diameter (LAD) was found to have an AUC generally over 0.7 in AF patients, with relatively balanced specificity and sensitivity. One study about low-score patients reported an AUC of 0.878 (cutoff point 43.5 mm, sensitivity 84.4%, specificity 74.7%), and if we bind LAD with CHA2DS2-VASc, we were still able to obtain a much better predictive value (AUC 0.747 vs. 0.541) (19, 31, 34, 36, 51). Left atrium top and bottom diameter (LTD) also has a decent value (AUC 0.705, sensitivity 75.7%, specificity 73.7%), which is not as good as LAD.

**FIGURE 2:**
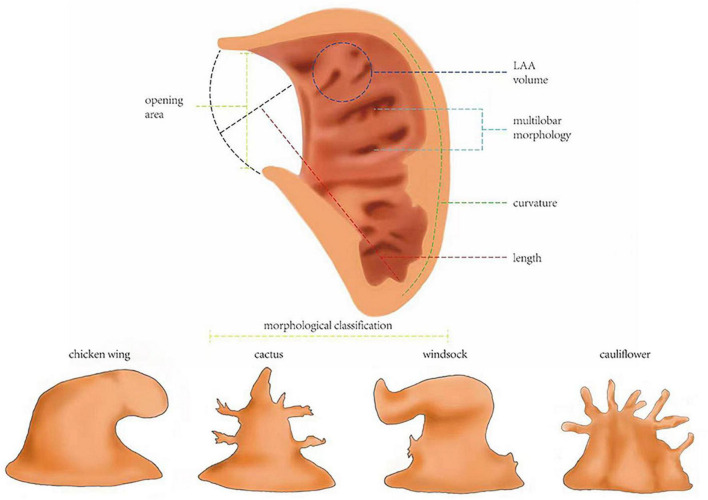
Definitions of the morphological indicators. Morphological classification: left atrial appendages (LAAs) are divided into four classical types, namely, chicken wing type, cactus type, windsock type, and cauliflower type. Multi-lobar morphology: having several lobules itself is a risk factor for thrombosis. Opening area: the area of the LAA orifice. LAA volume: including LAA end-systolic volume and LAA end-diastolic volume. Length: in the maximum diastolic state of the LAA, the length of the line from the top of the LAA to the midpoint of the junction point of the LAA and left atrial wall to that between the LAA and left upper pulmonary vein. Curvature: the degree to which the curve of LAA deviates from a straight line. Defined as L/D-1, where L is the centerline length and D is the Euclidean distance between the centerline endpoints.

In summary, although many further studies on quantitative morphological indicators are expected to be carried out to obtain a clear cutoff point, sensitivity, and specificity, they exhibited enough value to be highly expected to play a role in the prediction model (see Figure 2 for definitions of the morphological indicators).

### 3.2. Hemodynamic indexes and their predictive value for thrombosis

#### 3.2.1 Blood dynamics-related indicators and their value in thrombosis prediction

The abnormal shape and motor function of LAAs in patients with AF will beyond doubt affect the blood flowing inside and outside, causing thrombus formation, and blood flow-related indexes can rank among the most ideal predicting methods, with AUCs more than 0.8 (Table 1). Studies showed that the velocity of blood flow slows down in patients with AF due to the impairment of LAA’s systolic or diastolic function. When the blood flow velocity in the LAA is less than 40 cm/s, it is related to the higher risk of stroke (OR = 10.9 together with the LAA orifice area) (46). Blood flow velocity inside LAA was found to have an AUC of 0.799 in predicting atrial thrombosis (vs. 0.746 of CHA2DS2-VASc score), a sensitivity of 0.906, and a sensitivity of 0.702 at the point of 42.25 cm/s (23). If we subdivide the velocity into small parts, we can find their predictive value even better. The cutoff point of the average emptying velocity of LAA (LAA-AEV) to predict thrombus was 33.0 cm/s, and the specificity and sensitivity were 80.0 and 86.7%, bringing a high AUC of 0.887, while the point of the average filling velocity of LAA (LAA-AFV) was 27.5 cm/s, and the specificity and sensitivity were, respectively, 73.3 and 96.7%, and its AUC was as high as 0.882 (52). An LAA emptying velocity (LAA-FPV) < 55.4 cm/s was believed to be able to predict thrombus formation in patients with AF with an AUC of 0.869, a sensitivity of 85.7%, and a specificity of 82.8% (53). The AUC of LAA peak emptying velocity reached a height of 0.893 (48). Corresponding to the blood flow velocity, the time when the blood stays in the LAA and blood flow energy-related indicators are also ideal tools. If the average residence time in LAA is more than 2 s, a thrombus can be formed (54). These parameters all have the potential to be used in clinical practice; for example, the high sensitivity of LAD (96.7%) exhibits its potential to be used for the accurate prediction of patients’ relatively high risk when doctors are in doubt.

In addition to speed-related indexes, other blood flow-related indicators have similar high predictive values and should also be taken into consideration when building risk assessment models. The critical value of ejection fraction of LAA (LAA-EF) to predict the risk of thrombus in patients with AF was 21 and 20% reported by two groups, and the sensitivity and specificity of predicting LAA thrombus were 92 and 88%, respectively (55, 56). A new model including LAAEF showed an AUC of 0.886, which far exceeds that of the CHA2DS2-VASc score (0.579). Outside LAA, the ejection fraction of the left ventricle (LVEF) is another focus of attention. LVEF exhibited an OR of 4.38 (30). When tested among patients whose CHA2DS2-VASc = 0 or 1, its AUC reached 0.76, and when it is combined with the CHA2DS2-VASc scale, the AUC was 0.69 for all of the included patients with AF. It seems that it is not very high, but for the same group, the CHA2DS2-VASc score showed only an AUC of 0.59 (29). One group established a new predictive model with LAEF [predictive score = 1 (non-paroxysmal AF) + 2 (LVEF% ≤ 55%) + 3 (LAE)], and this model brought great promotion to accuracy (AUC 0.792 vs. 0.608 of CHA2DS2-VASc) (30). In addition to ejection fraction, the generation of the vortex can reflect morphological changes as well, and the position of the vortex core can also be used as a predictor. It was found through computer simulation that the vortex core of a healthy heart extends to the tip of the left atrial ear, leading to stronger blood flow erosion, which prevents thrombus formation, while in the pathological model with AF, the vortex current is obscure (16).

In conclusion, blood dynamics-related indicators have satisfying predictive value, with examples of new models built on them, whose performance was splendid, especially in patients with low-score AF, which makes us believe that they are definitely suitable supplements for CHA2DS2-VASc to improve the accuracy of individual risk forecasts.

#### 3.2.2 LAA dynamics-related indicators and their thrombosis prediction value

When we mention hemodynamic indexes, not only blood is involved but also the motion of the LAA itself. We found that LAA motion-related indexes also have adequate qualifications to be taken into the risk prediction model if further investigations can remove some barriers. In patients with AF, the motor function of the LAA is limited, and the LAA wall motion velocity (LAA-WMV), regardless of the systolic velocity (LAA-SV) or diastolic velocity (LAA-DV) of each wall of the LAA, was decreased, as was the area change rate of the LAA, which has a direct relationship with thrombosis formation. The velocity of the mitral annulus (e′) was also considered to be an independent index for predicting LAA thrombus in patients with AF (53). However, this study about these indicators stayed at a qualitative retrospective stage, and neither the exact predictive value nor the sensitivity and specificity were proposed. Some further studies and analyses are needed to find precise data for practical clinical usage.

**FIGURE 3:**
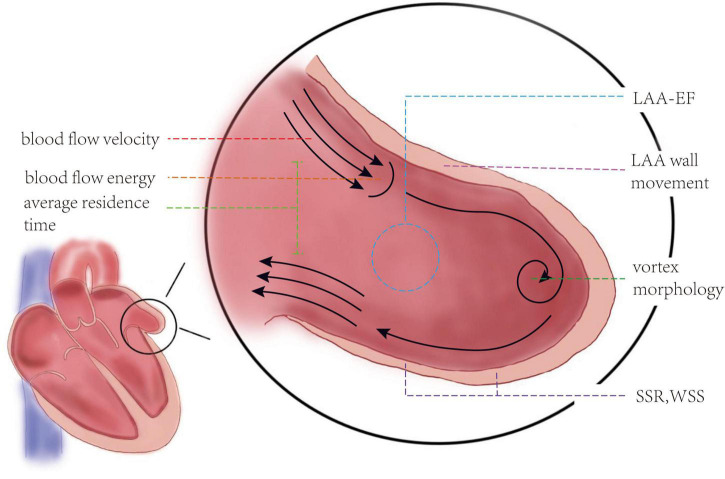
Definitions of the hemodynamic indicators. Blood flow velocity: the speed blood goes into or out of left atrial appendage (LAA). To study the relationship between blood flow velocity and thrombosis, it is divided into LAA-AEV, LAA-AFV, LAA-PEV, and LAA-FPV. Blood flow energy: the kinetic energy of blood flow. Average residence time: the time that blood stays in the LAA. LAA-EF: left atrial appendage ejection fraction, usually calculated by area change rate, LAAEF = (maximum area of LAA–minimum area of LAA)/maximum area of LAA. Vortex morphology: the shape of vortex blood flow, such as the location of the vortex core. LAA motion velocity: the systolic velocity and diastolic velocity of each wall of the LAA. SSR, systolic strain rate; the overall maximum negative strain rate in systole was taken as the SSR. WSS, wall shear stress; shear stress is defined as the component of stress coplanar with a material cross-section. WSS shows the friction between blood and LAA wall.

As the motion of blood and the structure of LAA both transform to some extent, what the blood will do to the LAA wall, which is to say, the strain-related indexes of LAA may also change accordingly. Therefore, we inspected the strain state of the LAA and conclude that these indexes have the potential to play a role in predicting thrombosis risk in patients with AF. There was an all-sided analysis of LAA 2D-strain, which found that, regardless of global, medial, apical, or lateral strain, they all have decent specificity and sensitivity (Table 1), and LAA minimum strain has impressive predictive value (AUC = 0.798) (5, 48). According to a sampling calculation, when the systolic global SSR of LAA is less than 2.80 s^–1^, the risk of thrombus formation in patients with AF can be predicted with a sensitivity of 75% and a specificity of 82.8% (AUC = 0.809) (53). Compared with the health model, the SSR of patients with AF decreased rapidly to zero from LAA’s opening to tip, where the blood was prone to stagnation and thrombosis, for if the SSR drops to 10 s^–1^, it can lead to a significant increase in blood viscosity (16). Moreover, a numerical simulation found that the shear force on the calculated example’s LAA wall was smaller than that of the sinus rhythm. In the early stage of contraction, there was no significant difference in the distribution of wall shear stress (WSS), but in the late contraction stage, the WSS value of patients with AF decreased significantly (10, 57). If the heart wall was fibrotic, the WSS of the fibrotic part of the LA wall would be lower (58). However, it is a pity that these studies only gave qualitative conclusion instead of moving forward a single step to use them in prediction, so we have yet to determine the exact value of WSS. Anyway, LAA strain-related indexes, such as SSR, have good predictive value without doubt, while WSS also has the potential to become a practical prediction index as good as SSR, thus illustrating that the strain state is a possible excellent predictor of thrombus generation. The relatively higher specificity of SSR exhibits its ability to confirm that patients do not have such high risks, which is useful when we want to illustrate that anticoagulants are not that necessary in some patients (see Figure 3 for definitions of the hemodynamic indicators).

## 4. Limitations and future direction

### 4.1. The limitations of our study

If we can construct a personalized risk assessment system according to more direct morphological and hemodynamic indicators, it will be a great convenience for clinical work; but, at present, there are still many barriers. The morphological classification and evaluation are difficult to be replicated objectively. Although fractal geometry theory may be a good tool to evaluate the complexity and irregularity of LAA quantitatively, it needs special examination and calculation, which is still too complex for daily use. As to the quantitative morphological and hemodynamic indexes, although most of them mentioned above have clear, comparable predictive value, it is still hard to say which index is definitely better considering the difference in baseline data of each study. Taking the differences in AF type as an example, patients with persistent AF were reported to have an LA thrombosis rate of up to 6–18%, which is significantly higher than that of patients with paroxysmal AF, and the brain embolism rate of elder patients with paroxysmal AF (2.7%/year) was also reported to be significantly lower than that of elder patients with persistent AF (5.1%/year) (59, 60). In other words, although our review shows that some of the parameters, such as LAD and blood flow velocity-related parameters, have higher AUCs, the conclusion may not be correct. Additionally, although our review mainly focused on thrombosis risk and the endpoint of many studies included may be LA/LAA thrombus, which is the main cause of stroke in AF (61), doctors may care more about predicting specific thrombosis events, and in our study, only LAA orifice area, LAD, and LAA flow velocity were reported to be used in predicting thromboembolic events like stroke (34, 46), so some more direct studies are needed. Moreover, we found that many of the parameters reported above do not have an independent relationship with thrombus risk, and some of them may compensate for each other. To build an ideal predictive model, it may be necessary to take so many indicators together and view them comprehensively. For example, a lower swirl range or blood flow velocity and more than two lobules and a large LAA volume in patients with AF have a relatively higher risk of thrombosis, while a complex LAA with seven lobules was affected by other factors, such as opening diameter and volume, and reached the lowest risk of coagulation among all study cases (40, 62). What we will worry about is that, even though we ultimately find the exact indicators for risk prediction, the comprehensive analysis of that multi-index model is still undoubtedly beyond men’s power.

### 4.2. Potential future directions

Although difficult to bring so many morphological and hemodynamic indicators into clinical usage, with the rapid development of information technology, we can build an online platform with the help of artificial intelligence (AI) for doctors with the need to calculate the scores for a precise individual thrombus risk in patients with AF, especially those with low CHA2DS2-VASc scores, which is most likely the future direction. A deep learning framework has already been established, attempting to train AI for real-time estimation of patients’ thrombus risk. They did not build the framework based on hemodynamic and morphological indicators, but the team stressed the need for more advanced risk indicators (43, 63) for whom the morphological hemodynamic ones discussed above may be a perfect fit.

Artificial intelligence may be a bright future and can be set as a long-term goal; but, at present, it is still too hard to build a new predictive model, let alone teaching computers to calculate for us, because another barrier for the application of morphological and hemodynamic parameters is that they cannot be easily got. The CHA2DS2-VASc score, though comes with some disadvantages, still keeps a great advantage called convenience, while most of the satisfying indexes mentioned above are acquired for imaging or ultrasonic examinations, such as TEE, which increase costs and are time-consuming, which beyond doubt cannot be operated on every patient. Therefore, at the moment, we hope that a short-term goal can be set as reaching a consensus that a few morphological or hemodynamic parameters can serve as a supplement to the CHA2DS2-VASc system in some specific cases, such as suspecting some patients with AF with low CHA2DS2-VASc scores of high thrombosis risks, to provide a basis for advising anticoagulant therapies for them.

## 5. Conclusion

In summary, it is an accurate and good method to predict the thrombus risk in patients with AF by reflecting the abnormal morphology of LAA through hemodynamic indexes. It has the advantages of quantitative objectivity and convenient clinical application and can be used for prediction. It has certain clinical value for reasonably evaluating the thrombus risk in patients with AF, especially those with low CHA2DS2-VASc scores, being a potential solution for the dilemma of determining the medication scheme of risky drugs, and selecting suitable patients for LAA occlusion. With the rapid development of information technology, it is expected to build an online platform based on AI to comprehensively analyze a variety of indicators and establish an evaluation system. It is believed that it will bring more convenience to clinical work and improve the quality of life of patients.

## Author contributions

YS, ZC, and ZW wrote main parts of the manuscript and produced graphics. YQ, YL, and TL reviewed and edited the manuscript. YS, ZC, and QT drafted the final version of the manuscript. All authors prepared the manuscript, read, and approved the final manuscript.
